# Microvascular Reactivity Measured by Dynamic Near-infrared Spectroscopy Following Induction of General Anesthesia in Healthy Patients: Observation of Age-related Change

**DOI:** 10.7150/ijms.52433

**Published:** 2021-01-01

**Authors:** Ah-Reum Cho, Hyeon-Jeong Lee, Hyae-Jin Kim, Wangseok Do, Soeun Jeon, Seung-Hoon Baek, Eun-Soo Kim, Jae-Young Kwon, Hae-Kyu Kim

**Affiliations:** 1 Department of Anesthesia and Pain Medicine, Medical Research Institute, Pusan National University Hospital, Busan, Republic of Korea.; 2 Department of Anesthesia and Pain Medicine, Pusan National University, School of Medicine, Yangsan, Republic of Korea

**Keywords:** Aging, cardiovascular physiology, anesthesia, inhalation, microcirculation, spectroscopy, near-infrared

## Abstract

**Background:** The purpose of this study was to investigate the effect of general anesthesia on microvascular reactivity and tissue oxygen saturation (StO_2_) using near-infrared spectroscopy in conjunction with vascular occlusion tests (VOT). Age-related changes of microvascular reactivity, that is, the capacity of capillary recruitment, were examined.

**Methods:** This prospective observational study was performed on 60 patients without comorbidities who underwent elective surgery under general anesthesia. Baseline StO_2_ on thenar eminence, hemodynamics, and laboratory profile were monitored before (T0) and 30 min after general anesthesia (T1). During VOT, occlusion slope representing oxygen consumption of muscle and recovery slope representing microvascular reactivity were also collected at T0 and T1.

**Results:** Baseline StO_2_ and minimum / maximum StO_2_ during VOT increased under general anesthesia. Occlusion slope decreased while the recovery slope increased under general anesthesia. To observe aging effect, Receiver operating characteristic analysis was performed and age less than 65 years old showed a fair performance in predicting the increase of microvascular reactivity after the induction of anesthesia (AUC 0.733, 95% CI 0.594-0.845, P= 0.003). For age-related analyses, 27 patients of younger group (< 65 years) and 26 patients of older group (≥ 65 years) were divided. Recovery slope significantly increased under general anesthesia in younger group (2.44 [1.91-2.81] % ∙ sec^-1^ at T0 and 3.59 [2.58-3.51] % ∙ sec^-1^ at T1, P <0.001), but not in older group (2.61 [2.21-3.20] % ∙ sec^-1^ at T0, 2.63 [1.90-3.60] % ∙ sec^-1^ at T1, P = 0.949).

**Conclusions:** General anesthesia could improve StO_2_ through increase of microvascular reactivity and decrease of tissue metabolism. However, microvascular reactivity to capillary recruitment under general anesthesia significantly improves in younger patients, not in older patients.

## Introduction

Microcirculatory dysfunction and impaired tissue perfusion may persist even if systemic hemodynamic variables are optimized and within the therapeutic target 1,2. This suggests that clinicians should identify therapeutic targets for functional microcirculatory restoration to prevent organ failure 3. Microcirculatory function is impaired in a number of pathologies, including sepsis 4, diabetes mellitus 5, shock 6,7, aging 8, and smoking 9. However, research on the effects of anesthetics on microcirculatory function has begun only recently, and the results are still inconsistent 10,11. One study reported that general anesthesia has a negative impact on reperfusion reserve 10. Another study showed that general anesthesia increases the recovery slope, suggesting improvement of tissue microvascular reactivity and peripheral vasodilation 11. As patients with microcirculatory dysfunction due to various underlying diseases were included in previous studies, inconsistent outcomes may have occurred. To accurately determine the effect of anesthetic agents on microvascular reactivity, patients without comorbidities must be studied. Moreover, age could also be an important consideration in studies on microcirculatory function, even in healthy subjects 12.

Owing to near-infrared spectroscopy (NIRS) technology, the detection of microcirculatory dysfunction has become easier and non-invasive. Many studies have shown that the dynamic NIRS parameters that can be measured with a brief ischemic challenge are clinically more useful than static tissue oxygen saturation (StO_2_) values 10,13-15. A vascular occlusion test (VOT) is a method of observing the change in StO_2_ after applying a tourniquet with a higher pressure than the patient's systolic blood pressure. The StO_2_ reduction rate (occlusion slope) during the ischemic period reflects oxygen extraction. The rate of StO_2_ increase (recovery slope) during reperfusion after the release of the vascular occlusion reflects microvascular reactivity 10,13,14.

The purpose of this study was to investigate the effect of anesthetics on microvascular reactivity and StO_2_ using near-infrared spectroscopy in conjunction with VOT. Age-related changes of microvascular reactivity, which means the capacity of capillary recruitment, were focused.

## Methods

### Patients

This study was approved by the Institutional Review Board of Pusan National University Hospital (IRB no. 1607-012-054, Busan, South Korea) and written informed consent was obtained from all subjects participating in the trial. The trial was registered prior to patient enrolment at clinicaltrials.gov (NCT03060798, Hyeon-Jeong Lee: February 19, 2017). This manuscript adheres to the applicable STROBE statements.

Sixty adult patients who were referred for elective surgery under general anesthesia participated in this study. Exclusion criteria were an American Society of Anesthesiologists (ASA) physical status classification > II, age < 18 or > 80 years, body mass index > 30 kg m^-2^, disorders likely to influence microcirculation (uncontrolled hypertension, diabetes, peripheral vascular disease, or chronic venous insufficiency), pregnancy, smoking, sleep apnea, history of chronic obstructive and restrictive pulmonary disease, contraindications for anesthetic agents, or refusal to participate in the investigation.

### General anesthesia

In the operating room, standard monitoring including electrocardiography, non-invasive blood pressure (NIBP) monitors, and pulse oximetry were used. Skin temperature probes (400 series, GE Healthcare, Helsinki, Finland) were placed on the right palm. The operating room temperature was maintained at 22-24°C. Skin temperature was measured before and after induction of anesthesia. General anesthesia was induced with propofol 1.5 - 2 mg ∙ kg^-1^ and a continuous infusion of remifentanil 0.2 μg ∙ kg^-1^ ∙ min^-1^. After injection of rocuronium 0.8 mg ∙ kg^-1^, tracheal intubation was performed and mechanical ventilation was applied with an inspired oxygen fraction of 0.5, a tidal volume of 8 ml ∙ kg^-1^, and a respiratory rate adjusted to maintain end-tidal CO_2_ of 30-35 mmHg. General anesthesia was maintained with desflurane and remifentanil to maintain a bispectral index (BIS; Bispectral Index™, Aspect Medical System, Norwood, MA, USA) of 40-50 and hemodynamic parameters within 20% of the baseline values. Hartman's solution was infused during the study period at the rate of 5 ml ∙ kg^-1^ ∙ h^-1^. Under general anesthesia, blood samples were collected to measure the serum lactate level.

### Vascular occlusion test (VOT)

VOT was performed twice for each patient, before (T0) and 30 min after the induction of general anesthesia (T1). Before induction, a NIRS sensor (INVOS^TM^ 5100C Cerebral/Somatic Oximeter; Medtronic, Minneapolis, MN, USA) was placed on the thenar eminence and an automated tourniquet (A.T.S^®^ 3000 Automatic Tourniquet System; Zimmer Inc., Warsaw, IL, USA) was placed around the ipsilateral upper arm. A NIBP was placed around the contralateral upper arm and the baseline blood pressure was measured. When the baseline StO_2_ was stabilized, the automatic tourniquet was inflated to 50 mmHg over the patient's baseline systolic blood pressure and maintained for 5 min. After the 5 min ischemic period, the tourniquet was rapidly deflated to 0 mmHg. StO_2_ data were continuously recorded during the VOT procedure. Baseline StO_2_, minimum StO_2_ during the 5 min inflation of the tourniquet, time to minimum StO_2_, maximum StO_2_ during deflation of the tourniquet, and time to maximum StO_2_ were obtained. The occlusion slope and recovery slope, which are VOT-derived dynamic parameters related to microcirculatory reactivity, were calculated based on the measured StO_2_ data. The occlusion slope was defined as the slope of StO_2_ descent to the lowest value. The recovery slope was calculated from deflation of the tourniquet until the recovery of StO_2_ to the highest value.

### Data collection

Preoperative hemoglobin concentration was obtained. Mean blood pressure (MBP), heart rate (HR), pulsed oxygen saturation (SpO_2_), skin temperature, and VOT-derived measurements, including baseline StO_2,_ occlusion slope, minimum StO_2_, time to minimum StO_2_, recovery slope, maximum StO_2_, and time to maximum StO_2_ were recorded at T0 and T1. Serum lactate level was measured at T1.

### Statistical analysis

The primary outcome of this study was the difference of the recovery slope of the VOT after the induction of general anesthesia. According to our pilot study performed prior to this investigation, mean difference of recovery slope of 0.28 % ∙ sec^-1^ with SD of 0.72 % ∙ sec^-1^ was obtained. A sample size of 54 patients was necessary to gain a power of 80%, by using a Wilcoxon signed rank test with a two-sided significance level of five. Considering a drop-out rate of 10%, a total of 60 patients were required.

Data are expressed as number (proportion), median [IQR], or mean (SD). All continuous variables were tested for normality assumption with a Q-Q plot and Kolmogorov-Smirnov test. StO_2_ values during VOT at T0 and T1 were compared with repeated measures ANOVA with a Bonferroni post hoc test. A paired t-test or Wilcoxon signed rank test was used to analyze differences in the hemodynamic variables and VOT-derived measurements between T0 and T1.

Patients were divided by an increase of recovery slope under general anesthesia and an unpaired student t test or Mann-Whitney U test was used to analyze differences in the patient's characteristics and hemodynamic variables between the groups. The significantly different variables were evaluated their ability to predict the increase of recovery slope of VOT under general anesthesia using a receiver operating characteristics (ROC) curves analysis with 95% confidence interval (CI). The optimal cut-off was selected to maximize the Youden index and age less than 65 years old showed a fair performance in predicting the increase of microvascular reactivity after the induction of anesthesia. For age-related analyses, patients were divided at 65 years old; 27 patients of younger group (< 65 years) and 26 patients of older group (≥ 65 years). An unpaired student t test or Mann-Whitney U test was used to analyze differences in the hemodynamic variables and VOT-derived measurements between the age groups.

A P-value < 0.05 was considered significant. All statistical analyses were performed using PASW Statistics for Windows, Version 18.0 (SPSS Inc., Chicago, IL, USA) and MedCalc for Windows, version 13.2 (MedCalc Software, Ostend, Belgium).

## Results

A total of 60 patients were enrolled in this study. Seven patients were excluded due to protocol violation. Demographic and intraoperative characteristics are summarized in Table 1.

### Receiver operating characteristic analysis to predict the increase of recovery slope under general anesthesia

**Figure 1:**
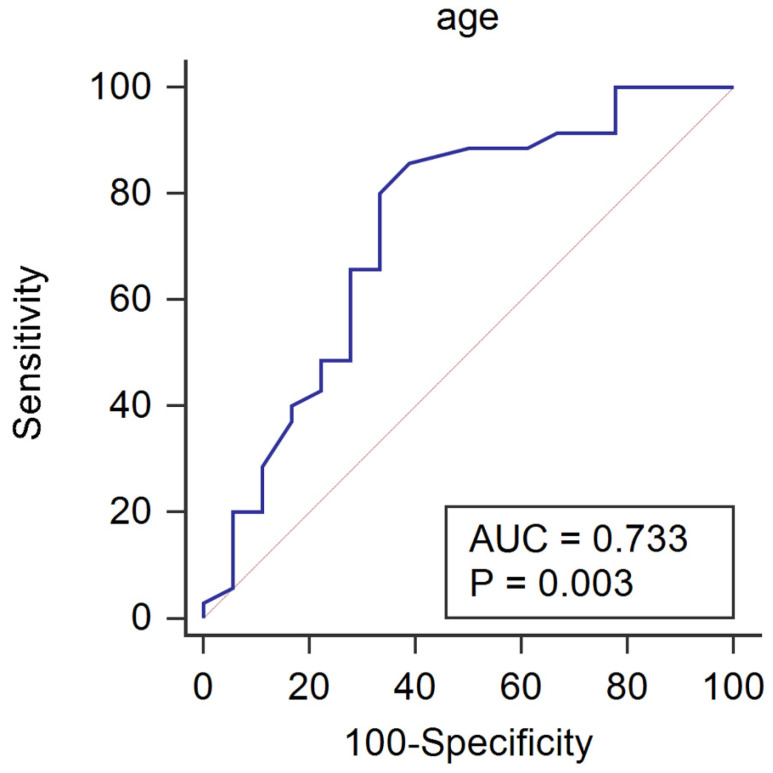
Receiver operating characteristic curve for age to predict the increase of recovery slope under general anesthesia. If age was lower than 65 years old, the increase of recovery slope under general anesthesia was predicted with a sensitivity of 85.7% and a specificity of 61.1%.

Patient's characteristics and hemodynamic variables of patients with and without the increase of recovery slope under general anesthesia are shown in Table 1. Patients whose recovery slope increased under general anesthesia were significantly younger than those whose recovery slope did not increase (P=0.013). The area under the ROC curve for age to predict the increase of recovery slope after the induction of anesthesia was 0.733 (Fig. 1, P = 0.003; 95% CI 0.594 - 0.845). If age was lower than 65 years old, the increase of recovery slope under general anesthesia was predicted with a sensitivity of 85.7% and a specificity of 61.1%.

### Effects of general anesthesia on VOT-derived parameters and hemodynamic variables

**Figure 2:**
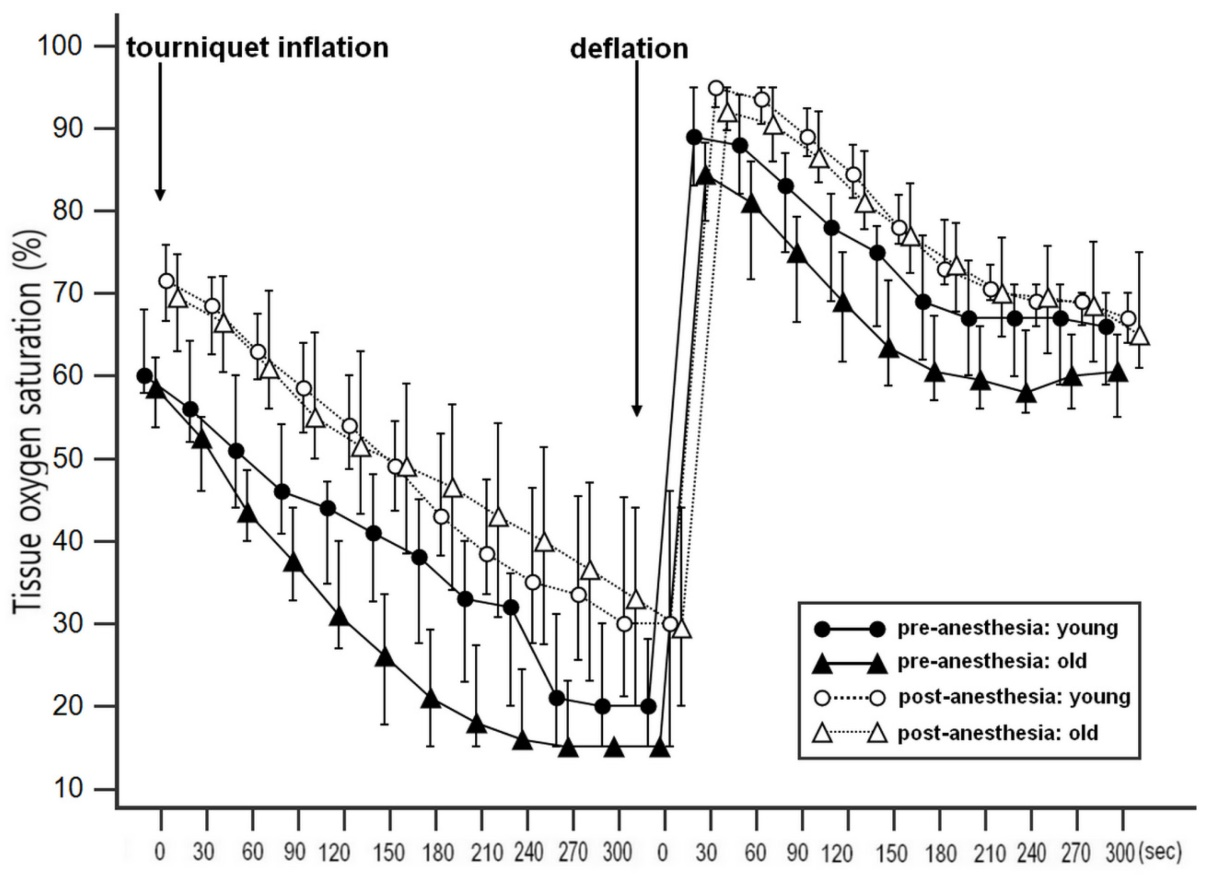
Changes in tissue oxygen saturation (StO_2_) during vascular occlusion test (VOT) before and after induction of general anesthesia in the younger and the older groups. A repeated measures ANOVA revealed significantly different StO_2_ values between before and after the induction of anesthesia, with a significant Bonferroni post hoc test for the differences at all the time points.

Demographic and intraoperative characteristics of two age groups divided by age of 65 years old are shown in Table 2. The type of operation and hemoglobin showed significantly different between the groups. The changes in StO_2_ during VOT before and after the induction of anesthesia in two age groups are shown in Fig. 2. Repeated measures of ANOVA revealed significantly different StO_2_ values before and after the induction of anesthesia in both groups (all p < 0.001); the Bonferroni post hoc test revealed significant differences at all time points. The changes in microcirculatory and hemodynamic parameters during VOT at T0 and T1 depending on the age are shown in Table 3. At T0, baseline StO_2_ was significantly higher in the younger group compared to the older group (P = 0.025). After the induction of anesthesia, recovery slope were significantly higher in the younger group compared to the older group (3.59 [2.58-3.51] % ∙ sec^-1^ in the younger group and 2.63 [1.90-3.60] % ∙ sec^-1^ in the older group, P = 0.047). MBP significantly increased and HR and skin temperature significantly decreased under general anesthesia in both groups. However, there were no differences in MBP, HR, SpO_2_, and skin temperatures between two age groups.

## Discussion

Our study showed that general anesthesia improves StO_2_ in healthy adult patients through an increase of microvascular reactivity and reduced metabolic demand. However, the important finding of our study was that the recovery slope significantly increased after the induction of general anesthesia only in the younger patients. This result suggests that general anesthesia may increase the microvascular reactivity and capillary recruitment only in the young, healthy patients 11,14. Concordantly, age could predict the increase of recovery slope after the induction of anesthesia, showing an area under the ROC curve of 0.733.

The recovery slope during VOT reflects the capacity of microvascular reactivity to recruit the capillary network 16. At rest, only 30% of capillaries are normally perfused, and 70% are not 17. These blood vessels are reserves that can be opened under stress conditions through relaxation of the precapillary sphincter and arteriolar vasodilation 18. Even in elderly patients without comorbidities in daily life, the limit of physiologic reserve can be seen during general anesthesia. This limited physiologic reserve is one of the causes of microcirculation changes during aging 19. In aged organs, dynamic control of the precapillary sphincter to regulate blood flow does not work well, and reserved vessels are barely recruited 20,21. A variety of mechanisms are involved, in which aging is associated with endothelial dysfunction, impaired vasodilatory and vasoconstrictive responses, increased vascular stiffness, and decreased vascular density and impaired vascular organization 8,22,23.

Vasodilation occurs in a complex way through endothelium dependent vasodilation (EDV) mediated by endothelium or endothelium independent vasodilation (EIV) mediated by smooth muscle of the vessel wall 12. EDV is known to decrease with age 12,24 and mechanisms include decreased NO sensitivity and bioavailability, and an increased oxidative stress 25,26. On the contrary, EIV is maintained even in aged vessel 27. Recovery slope of VOT has been known as a useful clinical tool for evaluating EDV 28,29. In our study, the baseline StO_2_ is much lower in elderly patients, however, other VOT-derived parameters were not significantly different at T0. Assuming EIV is age independent, EDV may be also preserved during VOT in older patients under non-stress condition. Inhalational anesthetic agents inhibit EDV but promote EIV via reduction of intracellular Ca^2+^ availability and sensitivity to contractile proteins 30,31. The net effect of these 2 opposing effects is generally vasodilation. Propofol and opioids decreases systemic vascular resistance in a predominantly EIV and partly mediated by EDV 32-34. Considering VOT at T1 was performed 30 min after the injection of propofol and remifentanil, mainly desflurane affected the microcirculation. Our results showed opposite results that desflurane promoted EDV in younger patients. The reason of the contradictory results is not elucidative, but may not be consistent with the in vitro results because numerous factors affect the microvascular system in vivo.

VOT parameters after the induction of anesthesia are not consistent among previous studies that showed a reduced or an increased recovery slope after the induction of anesthesia 10,11,35. We assume that the reason for these differences is that the previous studies included patients with various comorbidities who required cardiac surgery; our study included only patients without comorbidities. Recent studies showed that reactive hyperemia is significantly decreased in elderly patients with heart failure with preserved ejection fraction or hypertension compared to healthy age-matched controls, indicating endothelial dysfunction in these patient groups 36,37. Also, they demonstrated that vasodilation under loading condition, such as exercise, was blunted in these patients 36. Considering these results, patients who required cardiac surgery in the previous studies would have significant microvascular dysfunction and decreased capacity of reactive vasodilation. And microvascular dysfunction appears to be more severe in the cardiac patients than in heathy elderly patients.

There are numerous clinical variables that could contribute to regulate microcirculation in human. Therefore, it is difficult to confirm that the improvement of StO_2_ at T1 was solely due to general anesthesia. However, other potential factors, such as body temperature, stress hormone caused by intubation, fluid administration, and decreased MBP are estimated to have little influence on our results. Body temperature within normal range does not seem to influence StO_2_ 38. Previous study showed that stress hormones increased slightly in response to laryngoscopy and intubation, all returning to or below baseline 5 min later in normotensive patients 39. Moreover, 161 ml of Hartman's solution was infused in our study, which is not the amount that causes clinically significant hemodilution in people with normal hemoglobin. MBP was significantly decreased under general anesthesia where the lowest value was 66 mmHg which might provide enough perfusion pressure to allow peripheral autoregulation.

There are some limitations to the interpretation of the results of our study. First, VOT is not yet standardized, and the duration of peripheral ischemia is an area of strong debate. There are two methods to advocate peripheral ischemia: time-targeted and StO_2_-targeted VOT. We used 5-minute time-targeted VOT because a smaller minimum StO_2_ value after ischemia results in a more defined recovery slope 40,41. Second, it is difficult to confirm that older participants are completely healthy as younger patients. Because we enrolled only the patients undergoing minor surgery, more advanced hemodynamic monitoring or tests related to cardiovascular disease could not be performed. However, in clinical practice, completely healthy elderly is rare and most of the elderly have the compensated comorbidities in their daily life and more severe microcirculatory dysfunction, which more prominent results might be seen in real world. Finally, the effects of FiO_2_ on microcirculation are in debate. Previous study showed that normobaric hyperoxia reduced capillary perfusion and oxygen consumption and increased heterogeneity of the perfusion, while there was no change in recovery slope of VOT in healthy subjects 42. In the same line, high FiO_2_ had only a relatively small increase in mean (SD) StO_2_ of 5.3 (7.1) % and this effect reached a plateau when FiO_2_ was between 30% and 40% 43. When previous studies that investigated the effect of anesthetics on StO_2_ in cardiac surgery, StO_2_ under general anesthesia seems more likely affected by anesthetic agents, rather than FiO_2_ 9,11,35. In their studies, despite of increasing FiO_2_ to 0.5-0.6 under general anesthesia, only sevoflurane significantly increased StO_2_ and propofol did not significantly change StO_2_. Further research on the comparisons of effects of different types of general anesthetics on microcirculation of patients without underlying disease is needed.

General anesthesia is known to reduce cardiovascular function, which would ultimately reduce organ perfusion. However, our study shows that general anesthesia would compensate tissue hypoperfusion by improving microvascular reactivity representing the ability to recruit capillaries and reducing metabolic demands. However, improved microvascular reactivity by general anesthesia was only apparent in the younger patients without accompanying diseases, not in the older patients, even those without comorbidities. These findings suggest that general anesthesia could have different effects on microvascular reactivity depending on the status of the microvasculature. Additional studies on the effects of general anesthesia on microvascular reactivity in certain diseases are necessary.

## Figures and Tables

**Table 1:** Patient's characteristics and hemodynamic variables between patients with or without the increase of recovery slope under general anesthesia

	All patients (n=53)	Patients without the increase of recovery slope under general anesthesia (n=18)	Patients with the increase of recovery slope under general anesthesia (n=35)	P value
Age; years	56.5 (14.5)	63.3 (14.4)	53.0 (13.5)	0.013
Sex				0.145
Male	24 (45.3)	10 (55.6)	14 (40.0)	
Female	29 (54.7)	8 (44.4)	21 (60.0)	
Body mass index; kg.m^-2^	24.0 (3.2)	23.8 (3.4)	24.1 (3.1)	0.803
Weight; kg	63.1 (12.0)	62.8 (10.5)	63.2 (12.9)	0.906
Height; cm	161.6 (9.1)	162.3 (8.8)	161.3 (9.3)	0.713
ASA class				0.065
I	26 (49.1)	10 (55.6) 8 (44.4)15 (55.5)	17 (48.6)	
II	27 (50.9)	8 (44.4)	18 (51.4)	
Type of operation				0.301
Laparoscopic cholecystectomy	18 (34.0)	8 (44.4)	10 (28.6)	
Thyroidectomy	18 (34.0)	5 (27.8)	13 (37.1)	
Endoscopic sinus surgery	17 (32.1)	5 (27.8)	12 (34.3)	
Lactate; mg.l^-1^	0.99 [0.78-1.48]	0.79 [0.65-1.62]	1.02 [0.84-1.34]	0.296
Hemoglobin; g.dl^-1^	13.5 (1.5)	13.6 (1.4)	13.5 (1.6)	0.837
Fluid administration; ml	170.7 (29.7)	163.3 (28.9)	174.6 (29.8)	0.194
Mean blood pressure; mmHg		96.0 [86.5-112.5]	95.0 [89.3-104.5]	0.954
SpO_2_; %	99.0 [97.0-100.0]	99.0 [97.0-100.0]	99.0 [96.0-100.0]	0.512
Heart rate; beats.min^-1^	74.0 [64.0-86.0]	71.0 [60.0-76.0]	76.0 [66.0-88.0]	0.292
Skin temperature; °C	32.3 (2.3)	31.7 (1.7)	32.9 (1.9)	0.245

Data are number (proportion), mean (SD) or median [IQR].

**Table 2:** Patients' characteristics of the age groups

	Younger (< 65 years) (n = 27)	Older (≥ 65 years) (n = 26)	P value
Age; years	45.1 (10.7)	68.3 (5.8)	<0.001
Sex			0.502
Male	11 (40.7)	13 (50.0)	
Female	16 (59.2)	13 (50.0)	
Body mass index; kg.m^-2^	24.1 (3.3)	24.0 (3.1)	0.887
Weight; kg	64.2 (13.6)	62.0 (10.4)	0.521
Height; cm	162.7 (10.0)	160.5 (8.1)	0.389
ASA class			0.339
I	15 (55.5)	11 (42.3)	
II	12 (44.4)	15 (57.7)	
Type of operation			0.004
Laparoscopic cholecystectomy	5 (18.5)	13 (50.0)	
Thyroidectomy	8 (29.6)	10 (38.5)	
Endoscopic sinus surgery	14 (51.9)	3 (11.5)	
Lactate; mg.l^-1^	0.87 [0.78-1.18]	1.18 [0.78-1.58]	0.296
Hemoglobin; g.dl^-1^	13.9 (1.6)	13.0 (1.2)	0.031
Fluid administration; ml	176.4 (31.2)	165.4 (27.8)	0.278

Data are number (proportion), mean (SD) or median [IQR].

**Table 3:** Microcirculatory and hemodynamic parameters of the age groups.

	All patients (n=53)			Younger (< 65 yrs) (n = 27)			Older (≥ 65 yrs) (n = 26)		
	T0	T1	P value	T0	T1	P value	T0	T1	P value
Baseline StO_2_; %	60.0 [56.0-68.0]	70.0 [63.0-78.0]	<0.001	66.0 [60.0-68.8]	71.0 [62.5-77.8]	0.002	58.0* [53.0-65.0]	70.0 [63.0-78.0]	< 0.001
Occlusion slope; %.sec^-1^	0.19 [0.15-0.25]	0.15 [0.11-0.18]	<0.001	0.19 [0.16-0.25]	0.15 [0.12-0.18]	<0.001	0.21 [0.14-0.28]	0.12 [0.10-0.20]	< 0.001
Minimum StO_2_; %	15.0 [15.0-30.0]	29.0 [15.0-47.0]	<0.001	15.0 [15.0-31.5]	29.0 [15.0-46.0]	<0.001	15.0 [15.0-28.0]	27.5 [15.0-50.0]	<0.001
Time to minimum StO_2_; sec	271.0 [210.8-300.0]	300.0 [299.3-300.0]	<0.001	270.0 [214.3-300.0]	300.0 [297.8-300.0]	0.004	285.5 [210.0-300.0]	300.0 [300-300.0]	< 0.001
Recovery slope; %.sec^-1^	2.50 [1.99-2.97]	2.77 [2.24-3.98]	0.004	2.44 [1.91-2.81]	3.59 [2.58-3.51]	<0.001	2.61 [2.21-3.20]	2.63* [1.90-3.60]	0.949
Maximum StO_2_; %	87.0 [80.8-95.0]	95.0 [89.8-95.0]	<0.001	90.0 [82.5-95.0]	95.0 [90.5-95.0]	0.008	86.5 [79.0-91.0]	93.5 [89.0-95.0]	<0.001
Time to maximum StO_2_; sec	27.0 [23.8-30.5]	22.0 [16.8-28.3]	0.202	26.0 [20.5-34.3]	24.0 [15.5-32.5]	0.559	28.0 [23.0-34.0]	22.0 [18.0-30.0]	0.264
Mean blood pressure; mmHg	95.5 [87.0-107.0]	89.0 [78.0-100.0]	<0.001	94.5 [87.0-104.0]	88.0 [77.0-99.0]	0.003	97.5 [88.0-108.5]	89.0 [78.5-103.0]	0.003
SpO_2_; %	99.0 [97.0-100.0]	100.0 [98.0-100.0]	0.004	99.0 [97.0-100.0]	99.5 [98.0-100.0]	0.469	99.0 [96.0-100.0]	100.0 [98.0-100.0]	0.001
Heart rate; beats.min^-1^	74.0 [64.0-86.0]	82.0 [75.3-93.0]	<0.001	78.0 [66.3-87.8]	85.5 [77.0-98.0]	0.029	72.0 [59.5-78.5]	82.0 [75.0-90.3]	0.013
Skin temperature; °C	32.3 (2.3)	33.5 (1.99)	<0.001	31.9 (1.8)	33.4 (2.1)	<0.001	32.3 (1.9)	33.6 (1.6)	0.041

Data are mean (SD) or median [IQR]. T0 = before the induction of anesthesia; T1 = after the induction of anesthesia; StO_2_ = tissue oxygen saturation. *p < 0.05 compared to the younger group.

## Abbreviations

ASA: American Society of Anesthesiologists
BIS: Bispectral index
CI: Confidence interval
EDV: Endothelium dependent vasodilation
EIV: Endothelium independent vasodilation
HR: Heart rate
MBP: Mean blood pressure
NIRS: Near-infrared spectroscopy
SpO2: Pulsed oxygen saturation
ROC: Receiver operating characteristics
StO2: Tissue oxygen saturation
VOT: Vascular occlusion test
